# How Predictable Is the Operative Time of Laparoscopic Surgery for Ovarian Endometrioma?

**DOI:** 10.1155/2015/702631

**Published:** 2015-08-31

**Authors:** Pietro Gambadauro, Vincenzo Campo, Sebastiano Campo

**Affiliations:** ^1^Karolinska Institutet, LIME/NASP-C7, 17177 Stockholm, Sweden; ^2^Department of Obstetrics and Gynaecology, Catholic University of the Sacred Heart, Largo A. Gemelli 8, 00168 Rome, Italy

## Abstract

Endometriosis is a tricky albeit common disease whose management largely relies on laparoscopy. We have studied the operative times of laparoscopic endometrioma surgery in order to assess their predictability and possible predictors. One hundred forty-eight laparoscopies were included, with a median operative time of 70 minutes (mean 75.14; 95% CI: 70.03–80.24). Half of the cases had a duration within 15–20 minutes above or below the median (IQR: 55–93.75), but the whole dataset ranged from 20 to 180 minutes, and the standard deviation was relatively large (31.4). Surgical times were significantly related to technical (number and size of the cysts) and nontechnical factors (age, parity, dysmenorrhea, and family history). At multiple logistic regression, after adjusting for number and size of the cysts, surgical times below the first quartile were associated with older age (>30 years old: aOR: 3.590; 95% CI: 1.417–9.091) and parity (≥1 delivery: aOR: 3.409; 95% CI: 1.343–8.651). Longer times, above the third quartile, were instead predicted by a familial anamnesis of endometriosis (aOR: 3.639; 95% CI: 1.246–10.627). Our findings indicate highly variable surgical times, which are predicted by unexpected nontechnical factors. This is consistent with the complexity of endometriosis and its treatment. Productivity and efficiency in endometriosis surgery should focus on the quality of healthcare outcomes rather than on the time spent in the operating theatres.

## 1. Introduction

Endometriosis is a tricky albeit common disease whose management still largely relies on laparoscopic surgery [1]. Surgical excision of ovarian endometrioma has positive effects on pain and on the chances of spontaneous conception in subfertile women [2]. However, endometrioma surgery is complicated by concerns about recurrence and ovarian reserve as well as by a relative lack of knowledge on the pathophysiology of the disease [3, 4].

Surgery also represents a major cost for public healthcare because of the valuable human, technical, and logistic resources needed to operate on any single patient. Frequently, the cost profile of surgery in public funded healthcare is also worsened by cascading factors such as delays, cancellations, and long waiting lists [5]. Therefore, the usage of the operating theatre (OT) is often under the spotlight of decision-makers [6]. In spite of evidence that a great proportion of the OT time is lost on nonsurgical activities [7, 8], most surgeons have probably felt the pressure to finish a procedure at the planned time, victims of the belief that operative times are predictable. Our need for certainty makes it difficult to question the often wrong but somehow necessary prediction of surgical procedures duration, which is the basis for OT daily planning [9].

Surgeons are naturally major determinants of surgical times, but no perfect predictive tool exists. In gynecological surgery, different operations require different times, but variability in the duration of the same intervention is also common [10]. Laparoscopic surgery for endometriosis, apart from being often advanced, is peculiar because of a double nature, diagnostic and operative. In fact, see-and-treat interventions can be considered the norm rather than an exception. Ovarian endometriomas, for instance, are typically diagnosed at ultrasound but they often coexist with adhesions and peritoneal implants which can only be seen at laparoscopy. Technical and anatomical factors are usually considered responsible for the duration of surgery, but it would clearly be interesting to know if and how the duration of endometriosis surgery is predictable.

In this study, we have analyzed the distribution of operative times from a series of laparoscopic removals of ovarian endometrioma, with a focus on possible predictive factors.

## 2. Materials and Methods

We performed an analysis of operative times from a database including 148 cases of laparoscopic removal of ovarian endometriomas at the Department of Obstetrics and Gynecology of the Catholic University of the Sacred Heart, Rome, Italy. Our database was created for a study on endometrioma recurrence published by our group in 2014 [11]. Only elective cases of laparoscopic excision of histologically confirmed endometriomas ≥2 cm in diameter were included, while cases with deep endometriosis were excluded. The cystectomy was always performed by means of laparoscopic stripping of the cystic capsule after careful identification of the cleavage plane. Laparoscopy was performed under the care of one experienced laparoscopic surgeon (SC) and in a standardized fashion, as elsewhere described by Campo et al. [11]. All patients gave their informed consent in written form preoperatively.

In order to identify factors affecting the length of surgery, skin-to-skin operative times were analysed statistically together with other anonymized data. The distribution of operative times was first analyzed by descriptive statistics. Operative times were then evaluated in bivariate analysis together with several anamnestic and clinicosurgical variables. Correlation between surgical time and continuous variables such as age, BMI, cysts number, and largest diameter (cm) was studied by Spearman's rho. Association between categorical variables and surgical time was assessed by Mann-Whitney *U* test or Kruskal-Wallis one-way analysis of variance. Variables of epidemiological and anamnestic interest included dysmenorrhea, parity (≥1 delivery), infertility, and family history of endometriosis. The following categorical variables of technical interest were also considered: cyst number (single/multiple); cyst size (largest diameter ≤5 cm/>5 cm); cyst location (right/left/bilateral); peritoneal implants and adhesions; and intraoperative spillage of cystic contents.

Furthermore, we aimed at identifying factors that could be associated with procedures which are either shorter or longer than the expected time according to measures of central tendency, such as the median. Hence, we divided the cases into three groups depending on surgical times. The reference group consisted of cases with a surgical time comprised within the first and the third quartiles (Q1–Q3), while the other two groups consisted of cases, respectively, below the first quartile (<Q1) and above the third quartile (>Q3). Separate comparisons between the reference group and the other two groups were carried out by Mann-Whitney *U* test for continuous variables and chi-square test or Fisher's exact test for categorical variables. Postoperative hospital stay and complications were compared among the groups in a similar fashion. Variables showing a significant association with surgical times in the previous analyses (*p* < 0.05) were considered for simple and multiple logistic regression analyses. Crude and adjusted odds ratios (OR and aOR), with 95% confidence intervals (CI), were calculated to express the strength of associations between selected variables and surgical times. A *p* value of less than 0.05 was considered statistically significant. The statistical analysis was performed with SPSS Statistics (IBM) for Mac OSX and manually.

## 3. Results

One hundred forty-eight laparoscopic surgeries for ovarian endometrioma were included in analysis. No conversion to laparotomy was recorded. The median operative time was 70 minutes (mean: 75.14; 95% CI: 70.03–80.24). However, analysis of the distribution showed a high dispersion of the data. While 50% of the cases had a surgical time within 15–20 minutes above or below the median (interquartile range, IQR: 55–93.75), the whole dataset ranged from 20 to 180 minutes, and the standard deviation was relatively large (31.4).

When analyzing the entire dataset (Table 1), no significant correlation was found between surgical times and age, BMI, and cystic diameter. Instead, the surgical time was significantly and positively correlated with the number of cysts (*r* = 0.202; *p* = 0.014). The median surgical time was significantly longer when multiple rather than single cysts were removed (77.50 versus 70 minutes; *p* = 0.021). Anamnestic factors such as dysmenorrhea and nulliparity were also significantly associated with longer operative times. The operative time was not significantly affected by cyst location, adhesions, peritoneal implants, spillage, or infertility.

The cases were divided into three groups as previously described (Table 2). The reference group, serving as control, included 78 cases with an operative time ranging from the first quartile to the third quartile of the series (median: 70 minutes; IQR: 60–80). A short time group (<Q1) included 33 cases with a median of 40 minutes, while the long time group (>Q3) consisted of 37 cases with a median of 115 minutes. Compared to the reference group, women in the short time group were significantly older (median age: 34 versus 30; *p* = 0.018), and their cysts were more frequently single (87.9% versus 69.2%; *p* = 0.039) and smaller than 5 cm (87.9% versus 62.8%; *p* = 0.008). Significantly less women in the short time group were nulliparous but the number of infertile patients was similar in both groups. No differences were found in adhesions and peritoneal implants rate, but the absence of spillage was associated with shorter times (69.7% versus 44.9%; *p* = 0.017).

No statistically significant differences were found between the reference group and the longer operative time group except for a positive family history of endometriosis. Twenty-seven percent of women in the long time group had a family history of endometriosis compared to 8.9% in the reference group (10/37 versus 7/78; *p* = 0.011).

Crude odds ratios, with 95% confidence intervals and *p* values, describing the strength of association between selected variables and operative times, respectively, shorter and longer than the reference group (Q1–Q3) are presented in Table 3.

A multiple logistic regression analysis, adjusting for the number and size of the removed cysts, identified older age and parity as independent predictors of shorter operative times (Table 4). Longer operations were instead significantly associated with a positive familial anamnesis (aOR: 3.639; 95% CI: 1.246–10.627; *p* = 0.018).

Finally, while shorter operative times did not appear to improve short-term postoperative outcomes, belonging to the long operative time group was associated with a significantly longer postoperative hospital stay (>1 day for 35.1% of patients versus 15.4% in the reference group; *p* = 0.016) and a nonsignificantly higher postoperative complication rate (5.4% versus 0% in the reference group).

## 4. Discussion and Conclusions

Surgery, as a tool to treat medical conditions and improve quality of life, is invaluable. At the same time, costly operating theatres are a natural target of efficiency improving efforts [12].

In our study, we analysed the operative times of laparoscopic surgery for endometrioma in order to evaluate to what extent they are predictable. Since historical data and the surgeon's expert judgement are, in combination, the most common predictive approach, we focused on their respective assumptions: a limited variation of operative times and adequate knowledge of predicting factors.

Various conclusions can be drawn from our results. The first one is the fact that the time needed to perform a laparoscopic removal of ovarian endometrioma is highly variable and, as such, difficult to predict by simply looking at historical data. This might sound familiar to endometriosis surgeons, but similar findings have rarely been described by dedicated scientific studies [10]. Although half of the cases will last something within 15 minutes above or below the median time, a large proportion of the patients will have surgical times which are 50% or more shorter or longer. Endometriosis is certainly a complex disease, and this may reflect on the variability of operative times. Nevertheless, the cases in our study were quite homogeneous, since we only included patients with ovarian endometriomas ≥2 cm but excluded cases with elsewhere located deep endometriosis. Moreover, all the cases were performed under the care of an experienced laparoscopic surgeon, with a standardized stripping technique, and at the same institution, which is a reference centre for the treatment of endometriosis. Those conditions reduce the risk of performance bias, which is not uncommon in surgical research [13, 14].

A peculiarity of our study lies in the attempt to identify predictors of surgical duration for laparoscopic endometrioma surgery. Operative times are correlated to some of the surgical factors that we have analyzed, such as number and size of the cysts. However, other factors that might be considered to increase the surgical difficulty and to require additional time, such as adhesions and peritoneal implants, were not associated with a longer duration of the procedures. On the contrary, nontechnical factors such as age, parity, and family history were significantly associated with operative times. This further confirms the peculiar nature of endometriosis whose clinical manifestations and behavior change from patient to patient depending on factors so far largely unknown. For instance, the association of shorter times with older age or parity could be linked to a milder disease. Similarly, a positive family history for endometriosis, which we found to be associated with higher endometrioma recurrence rates [11], could be linked to a more severe disease and hence longer operative times.

Finally, shorter operative times do not seem to ensure short-term benefits in terms of postoperative stay or complications. This might depend on the fact that the overall operative times in this series were relatively low because of the team experience. On the other hand, patients whose surgical time was above the third quartile had a significantly longer postoperative stay, and the two postoperative complications of this series were found in this group. These findings cannot directly support a causal relationship and are limited by the sample size and a lack of controlling for possible confounders. However, they can be useful to formulate hypotheses on how to identify cases at risk at the time of surgery, allowing for tailored postoperative care planning.

Overall, our study highlights a new facet of the complexity of endometriosis and partially explains why it is difficult to predict operative times. The duration of laparoscopy is known to be less predictable than laparotomy [15], and this applies to different laparoscopic procedures [10]. Laparoscopy in endometriosis patients has a diagnostic value and often leads to see-and-treat management of unexpected findings [1]. Hence, strict time schedules are not convenient for endometriosis since they can lead to suboptimal surgery.

Besides, the heterogeneity of endometriosis seems to reflect also on the severity of the disease, for which we are still probably lacking important knowledge and a reliable staging system. Similar lesions, such as ovarian endometriomas, have variable pathological and clinical behaviors in different women [16], thus leading to surgical difficulty of unpredictable level or, as we have previously documented, different rates of recurrence [11].

Should we give up the efforts of making the operating theatre a more efficient place? Surely we should not do this, although proper targets should be identified.

To believe that surgical times, for any given operation, are standard and predictable according to historical measurements of central tendency such as the median, although tempting, is fallacious. The idea of standard procedures with standard times might satisfy our innate need for certainty and is also functional to other aspects of surgical planning, such as allocating instruments and staff [9]. However, because of the great dispersion of surgical times around the average values, scheduling according to historical averages is based on probability [9, 17]. The surgeons themselves, unfortunately, do not seem to be able to provide a more accurate estimate of operative times [18].

In this context, it would be reasonable to integrate predictive methods, based on historical data and the surgeon's estimate, with knowledge of the inevitable variability in operative time [19]. Measuring and adapting to the variability in surgical durations should be a key process of modern operating theatre management.

At the same time, efforts should be made in order to minimize the consequences to the patients and optimize resources' usage in other ways. On one hand, patient turnover could be improved by monitoring the time needed by standard perioperative procedures and activities [7, 8, 20]. On the other hand, we should maybe rethink our context where productivity is increasingly measured in number of procedures rather than in health outcomes, particularly in the case of such complex disease. Probably, the care of endometriosis patients should be provided by centres where the underlying production philosophy is mature enough to shift from a simplistic focus on quantity to a more refined demanding, but certainly patient-centered, interest for quality.

## Figures and Tables

**Table 1:** Factors affecting the operative time of laparoscopic surgery for ovarian endometrioma.

Variables		*p* value
Continuous variables	Spearman's rho	

Age	−0.113	0.172
BMI	0.066	0.425
Number of cysts	0.202	0.014
Largest diameter	0.084	0.309

Categorical variables	Operative time^a^	

Dysmenorrhea		
Yes (97)	75 (60–97.5)	0.038
No (51)	65 (40–90)	
Parity		
Yes (42)	60 (40–82.5)	0.020
No (106)	75 (60–95)	
Infertility		
Yes (35)	70 (60–90)	0.606
No (113)	70 (55–95)	
Positive family history		
Yes (20)	92.5 (56.25–110)	0.120
No (128)	70 (55–90)	
Cyst location		
Right (44)	60 (46.25–95)	0.258^b^
Left (73)	75 (50–90)	
Bilateral (31)	75 (60–100)	
Bilateral cysts^c^		
Yes (31)	75 (60–100)	0.570
No (13)	95 (57.5–115)	
Adhesions		
Yes (100)	72.5 (55–95)	0.093
No (48)	60 (45–90)	
Peritoneal implants		
Yes (62)	70 (55–91.25)	0.719
No (86)	70 (55–95)	
Spillage		
Yes (68)	72.5 (56.25–90)	0.564
No (80)	70 (45–95)	

^a^Operative times, in minutes, are presented as medians and interquartile range (IQR).

Comparisons for categorical variables were by Mann-Whitney *U* test.

^b^Calculated with Kruskal-Wallis test.

^c^Calculated only for cases with multiple cysts.

**Table 2:** Comparison between groups of cases with shorter, average, and longer operative time.

	Short time group <Q1	Reference group Q1–Q3	Long time group >Q3
Number of cases	33	78	37
Operative time (min)	40 (30–45)	70 (60–80)	115 (100–132.50)
Continuous variables			
Age (years)	34 (28.50–43)^a^	30 (27–35)	30 (25–37.50)
Number of cysts	1 (1-1)^a^	1 (1-2)	1 (1-2)
Largest diameter (cm)	4.0 (3.0–5.0)^b^	5.0 (4.0–6.0)	5.0 (3.0–6.75)
Categorical variables			
Older age (>30)	23 (69.7)^c^	36 (46.2)	18 (48.6)
Single cyst	29 (87.9)^c^	54 (69.2)	21 (56.8)
Small cysts (≤5 cm)	29 (87.9)^c^	49 (62.8)	23 (62.2)
Dysmenorrhea	17 (51.5)	52 (66.7)	28 (75.7)
Parity (≥1)	15 (45.5)^c^	18 (23.1)	9 (24.3)
Infertility	7 (21.2)	20 (25.6)	8 (21.6)
Positive family history	3 (9.1)	7 (9)	10 (27)^c^
Cyst location			
Right	12 (36.4)	19 (24.3)	13 (35.1)
Left	19 (57.6)	39 (50)	15 (40.5)
Bilateral	2 (6.1)	20 (25.6)	9 (24.3)
Bilateral cysts^d^	2 (50)^d^	20 (83.3)^d^	9 (56.3)^d^
Adhesions	20 (60.6)	52 (66.7)	28 (75.7)
Peritoneal implants	13 (39.4)	34 (43.6)	15 (40.5)
Spillage	10 (30.3)^c^	43 (55.1)	15 (40.5)
Hospital stay > 1 day	6 (18.2)	12 (15.4)	13 (35.1)^c^
Complications	0 (0)	0 (0)	2 (5.4)

Values are given as medians (IQR) for continuous variables or *n* (%) for categorical variables.

Short time and long time groups have been, respectively, compared with the reference group. All differences are nonsignificant (*p* ≥ 0.05) except for ^a^ *p* < 0.05 at Mann-Whitney *U* test, ^b^ *p* < 0.01 at Mann-Whitney *U* test, and ^c^ *p* < 0.05 at chi-square test.

^d^Calculated only for cases with multiple cysts.

**Table 3:** Factors associated with shorter or longer duration of laparoscopic surgery for ovarian endometrioma.

Variables	Shorter time <Q1			Longer time >Q3		
	OR	95% CI	*p* value	OR	95% CI	*p* value
Older age (>30)	2.683	1.129–6.377	0.025	1.105	0.505–2.419	0.802
Dysmenorrhea	0.531	0.232–1.217	0.531	1.556	0.641–3.774	0.329
Parity (≥1)	2.778	1.170–6.592	0.021	1.071	0.428–2.681	0.883
Positive family history	1.014	0.246–4.189	0.984	3.757	1.298–10.872	0.015
Single cyst (versus multiple)	3.222	1.020–10.183	0.046	0.583	0.260–1.310	0.192
Small cyst (≤5 cm)	4.291	1.370–13.440	0.012	0.972	0.434–2.180	0.946
Spillage	0.354	0.149–0.841	0.019	0.555	0.251–1.227	0.146

Crude odds ratios, with 95% confidence intervals and *p* values, describing the strength of association between selected variables and operative times, respectively, shorter and longer than the reference group (Q1–Q3).

**Table 4:** Nontechnical predictors of shorter and longer duration of laparoscopic surgery for ovarian endometrioma.

Variables	Shorter time <Q1			Longer time >Q3		
	aOR	95% CI	*p* value	aOR	95% CI	*p* value
Older age (>30)	3.590	1.417–9.091	0.007	1.009	0.453–2.249	0.983
Parity	3.409	1.343–8.651	0.010	1.031	0.408–2.605	0.948
Family history	1.304	0.298–5.707	0.724	3.639	1.246–10.627	0.018

Multinomial logistic regression analyses where the reference category of the dependent variable grouped laparoscopies with a surgical time between the first and third quartiles (Q1–Q3).

aOR: the odds ratios are adjusted for number of cysts (single/multiple) and largest diameter (≤5 versus >5 cm).
